# Characterization of Hydrogels of Gelatin/O-Carboxymethyl Chitosan in Ovule Form with Curcumin and Retinyl Palmitate as a Treatment for Cervical Cancer

**DOI:** 10.3390/gels12070618

**Published:** 2026-07-09

**Authors:** Melanie F. Ortega-Aguirre, Imelda Olivas-Armendariz, Juan C. Silva-Espinoza, Maryel E. Hernandez-Gonzalez, Laura E. Valencia-Gomez

**Affiliations:** 1 Instituto de Ingeniería y Tecnología, Universidad Autónoma de Ciudad Juárez, Ciudad Juárez 32320, Mexico; al203759@alumnos.uacj.mx (M.F.O.-A.); iolivas@uacj.mx (I.O.-A.); maryel.hernandez@uacj.mx (M.E.H.-G.); 2 Department of Biological Science, University of Texas at El Paso, El Paso, TX 79968, USA; jcsilvaespi@utep.edu

**Keywords:** gelatin, O-carboxymethyl chitosan, retinyl palmitate, curcumin, hydrogel, HeLa cells

## Abstract

At present, cervical cancer is the fourth most common cancer among women worldwide. Current treatments for cervical cancer, most notably chemotherapy, can induce various adverse side effects, such as nausea, vomiting, hair loss, and fatigue, among others. Therefore, alternative therapies are being developed. Intravaginal drug delivery systems based on biopolymers are the subject of extensive research for the treatment of this condition. Some of the most sought-after qualities in these systems are mucoadhesiveness, biocompatibility, and biodegradability, while ensuring controlled and prolonged drug release. Taking these aspects into consideration, this study develops hydrogel ovules composed of O-carboxymethyl chitosan (OCMC) and gelatin, crosslinked with genipin, containing retinyl palmitate at various concentrations, as well as curcumin. The characterization of functional groups and potential interactions between the polymeric matrix and the drugs was carried out by Fourier Transform Infrared (FTIR) spectroscopy. Physicochemical tests, including swelling, contact angle, and in vitro degradation assays, were also performed to determine the system’s hydrophilicity and its potential duration of use. In vitro release assays for both curcumin and retinyl palmitate were conducted to investigate the release kinetics of these two substances. To assess the mucoadhesiveness of the ovules, a sliding analysis was conducted using porcine uterine tissue. Finally, cell viability studies were performed using mouse fibroblasts and HeLa cells to evaluate the system’s specificity. The results of this study demonstrate that the system, composed of gelatin, OCMC, retinyl palmitate, and curcumin, is mucoadhesive, hydrophilic, and exhibits controlled swelling behavior. In vitro assays demonstrated that the system maintains control of both degradation and drug release for up to 72 h; moreover, it proved non-toxic to healthy cells, with the incorporation of curcumin shown to enhance the selective cytotoxicity against HeLa cells. Based on the foregoing, this drug delivery system containing retinyl palmitate represents a promising potential intravaginal treatment for cervical cancer.

## 1. Introduction

Cancer is a group of diseases that arises when cells proliferate uncontrollably; it can occur in any tissue and has multiple risk factors. Cervical carcinoma is one of the most prevalent cancers among women; some of its risk factors include infection with human papillomavirus (HPV), smoking, early sexual activity, multiple sexual partners (both for the woman and her partner), and multiple births [1]. In early-stage cervical cancer, small tumors are present in the cervix with little invasion beyond the uterus. In advanced stages of the disease, larger tumors are observed with extensive invasion into adjacent tissues and pelvic organs, often leading to metastasis. Depending on the stage of the disease, various treatments and combinations of treatments can be used, such as surgery, radiation therapy, and chemotherapy [2]. Among the most common chemotherapy drugs used to treat cervical cancer are cisplatin, topotecan, paclitaxel, nab-paclitaxel, gemcitabine, and 5-fluorouracil, among others, due to their antitumor effects. Chemotherapy is generally an aggressive treatment with substantial toxicity and a considerable decrease in the patient’s quality of life, which necessitates the search for new treatment strategies [3].

Curcumin is one of the secondary metabolites of the plant Curcuma longa, commonly known as turmeric. It is an active hydrophobic polyphenol, diferuloimelate, associated with treatments for various health conditions, including liver diseases, metabolic syndromes, and several types of cancer [4]. The anticancer potential of curcumin has been extensively investigated in a variety of cancer types. In the case of cervical cancer, curcumin has been shown to interfere with tumor development and progression through multiple mechanisms, including the induction of apoptosis, inhibition of cell proliferation, suppression of metastasis, and modulation of signaling pathways involved in tumor growth and survival. However, the clinical application of curcumin is limited by several drawbacks, including its poor aqueous solubility, low bioavailability, and limited absorption into the bloodstream following oral administration. These challenges significantly reduce their therapeutic efficacy. Consequently, considerable research efforts have focused on the development of novel curcumin-based formulations and drug delivery systems aimed at enhancing its stability, bioavailability, and antitumor activity for cancer treatment [5]. Some of curcumin’s anticancer mechanisms include the induction of apoptosis in HeLa cells via DNA damage, chromatin condensation, and the generation of reactive oxygen species (ROS) [6]. The inhibition of tumor cell proliferation by curcumin has been found to promote G2/M cell cycle arrest by activating apoptosis in a subpopulation of HeLa cells in the G1 phase [7]. A third mechanism suggests that high-dose curcumin inhibits metastasis and tumor angiogenesis [8]. An HPV infection mechanism where curcumin was found to be more cytotoxic to cervical cancer cells infected with HPV16 and 18 compared to cells not infected by the virus [9]. Finally, it has been reported that curcumin can induce autophagy and apoptosis [10].

Vitamin A and its derivatives (retinoids) play essential roles in the regulation of cell proliferation, differentiation, apoptosis, immune responses, epithelial integrity, collagen synthesis, and angiogenesis. These biological activities have attracted considerable interest for applications in tissue regeneration and the treatment of epithelial disorders [11], including premalignant and malignant lesions [12]. Several studies have reported that retinoids can modulate the progression of cervical intraepithelial neoplasia by promoting epithelial differentiation and regulating abnormal cell growth. Retinyl palmitate, a highly stable storage form of vitamin A, acts as a precursor of biologically active retinoids and has been widely incorporated into biomaterials due to its favorable stability profile [13]. Therefore, its incorporation into the present cervical film formulation was intended to provide local retinoid activity, supporting epithelial homeostasis and tissue remodeling while potentially contributing to the management of cervical lesions.

Vitamin A is a molecule involved in various cellular metabolic processes, such as cell differentiation and proliferation, immunity and epithelial integrity, collagen synthesis, and angiogenesis. Due to these qualities, it has been utilized in biomaterials applied to wound healing. Retinyl palmitate is the most stable form of retinoic acid (Vitamin A); in cosmetology, it is employed as an anti-aging agent due to its capacity to regulate the growth and differentiation of epithelial cells, as well as collagen production.

O-carboxymethyl chitosan (OCMC) is a derivative of chitosan obtained by incorporating carboxymethyl groups (-CH_2_-COOH) into the chitosan chain. Therefore, in addition to retaining several of chitosan’s properties, such as being biocompatible and antimicrobial, OCMC exhibits solubility across a wider pH range (pH > 6.0), allowing it to be dissolved in neutral solutions. This expands its potential for use in various biomedical applications compared to chitosan [14]. OCMC has been utilized in various types of biomaterials, most notably in wound dressings, tissue regeneration scaffolds, and matrices for controlled drug delivery [15].

Gelatin, a protein obtained through the hydrolysis of collagen, is one of the most widely used natural polymers worldwide, especially in the manufacture of biomaterials, owing to the abundance of functional groups -NH_2_, -COOH, and -OH within its structure [16]. Other advantages of gelatin include water solubility, lower cost compared to collagen, non-toxicity, biocompatibility, and biodegradability. However, it also presents several disadvantages, such as poor mechanical and thermal properties and a very short degradation time [17]. Hence, gelatin is typically employed in combination with other polymers to enhance its properties; for instance, it is used alongside chitosan in the fabrication of wound-healing films [18], mucoadhesive ovules for drug delivery [19], and electrospun scaffolds for applications in skin tissue regeneration [20].

Hydrogels are a form of controlled drug delivery system. Their structure consists of a polymer network linked via covalent and non-covalent bonds, as well as intermolecular interactions, and they contain a significant amount of water. Some of their key properties include high-water content, flexibility, and the ability to mimic the extracellular matrix environment of tissues [14]. Aside from that, to be considered suitable for a drug delivery system, hydrogels must be biocompatible (producing no toxic products during degradation), mechanically robust, capable of loading large quantities of a drug, able to provide controlled drug release, simple to manufacture, and easy for the patient to use [21]. Natural polymers are among the most widely used materials for the fabrication of hydrogels intended for medical applications, with gelatin, chitosan, and their derivatives being particularly notable examples, due to their versatility and compatibility with other materials, which enhances mechanical and chemical properties, using various cross-linking agents, including genipin [16]. Genipin is a natural chemical cross-linker derived from geniposide, which is an iridoid glycoside isolated from the fruits of the Genipa americana and Gardenia jasminoides plants. Previous studies have demonstrated that genipin is biocompatible when compared to other chemical cross-linkers, such as glutaraldehyde, formaldehyde, and certain epoxy compounds [21]. Based on these considerations, this study investigates the incorporation of retinyl palmitate into genipin-crosslinked gelatin/oxidized carboxymethyl chitosan (OCMC) hydrogels designed as curcumin-loaded vaginal ovules. The effects of retinyl palmitate on the physicochemical characteristics of the hydrogel system were evaluated, together with its capacity to reduce the cytotoxic effects associated with curcumin exposure in fibroblast cells. By combining the antitumor activity of curcumin with the regenerative properties of retinyl palmitate, this work aims to develop and validate a promising localized drug delivery platform for cervical cancer therapy.

## 2. Results and Discussion

### 2.1. Chemical Composition and Morphological Evaluation

**Figure 1:**
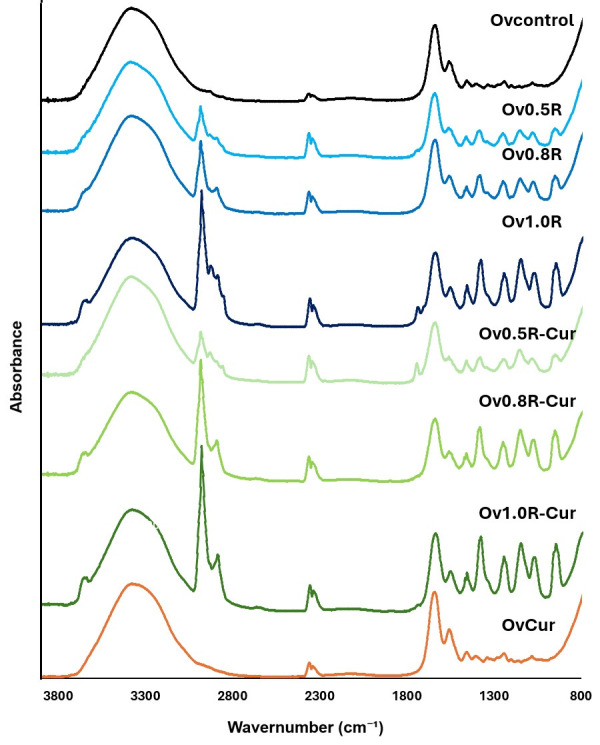
Spectra of the different formulations of hydrogels.

Figure 1 shows the spectra of the ovule formulations. For the OVcontrol, characteristic gelatin peaks were identified at 1567 cm^−1^, 1652 cm^−1^, and 3359 cm^−1^, corresponding to the C-N-H, C=O, and OH groups, respectively [18]. In the case of OCMC, characteristic bands were observed around 3400 cm^−1^ for the OH groups; a small peak at 2933 cm^−1^ for C–H; peaks at 1567 cm^−1^ and 3359 cm^−1^ for C-NH_2_; and peaks at 1652 cm^−1^ for C=O and 1445 cm^−1^. Regarding the carboxyl group, a slight peak was detected at 1469 cm^−1^ [22]. Interactions between gelatin and chitosan or OCMC have been reported in several studies, noting an intensified band around 3300 cm^−1^; this phenomenon is attributed to the formation of hydrogen bonds between the carboxyl groups of the gelatin and the amino groups of the chitosan or OCMC [19,22,23]. This intensified band is evident in all the formulations analyzed in the present study, appearing around 3359 cm^−1^, thereby confirming an interaction between the two polymers. This interaction indicates strong polymer–polymer molecular association, which contributes to the stabilization of the hydrogel structure. Finally, it has been reported that crosslinking by genipin in polymeric matrices of gelatin and chitosan or OCMC is evidenced by intensified bands at 1630–1640 cm^−1^ (amide C=O stretching vibrations) and 1540–1550 cm^−1^ (amine and amide N–H bending vibrations) [22,24]. These bands are observed in all formulations at 1567 cm^−1^ and 1652 cm^−1^, thereby confirming the crosslinking of the hydrogels, where genipin acts as a natural crosslinker stabilizing the hydrogel matrix.

No differences were found between the curcumin-loaded ovule (OVCur) and the control ovule (OVcontrol), as the characteristic bands of curcumin, located at 1628 cm^−1^ (C=C group), 3500–3200 cm^−1^ (OH group), and peaks at 1427 and 1512 cm^−1^ (attributed to C=O bond stretching, C–C–C bond vibrations, δC=O, and δC–H), are overlapped by the polymer bands [25]. This spectral overlap, together with the absence of significant shifts in the polymer backbone bands, suggests that curcumin is physically entrapped within the hydrogel matrix rather than chemically bonded.

In the case of the ovules containing retinyl palmitate (OV0.5, OV0.8, and OV1.0) and retinyl palmitate with turmeric (OV0.5-Cur, OV0.8-Cur, and OV1.0-Cur), the appearance of bands corresponding to the C–H stretching vibrations of the CH_3_ and CH_2_ groups is observed at 2979 and 2877 cm^−1^, respectively. The peaks in the region between 1467 cm^−1^ and 1164 cm^−1^ are assigned to the stretching vibrations of the –CO groups. Finally, the peak in the region of 1164 cm^−1^ was assigned to the carbonyl stretch. All these groups are characteristic of retinyl palmitate [26]; these signals, associated with aliphatic chains and ester functional groups, indicate successful incorporation of the lipophilic molecule into the hydrophobic domains of the polymeric matrix. Overall, FTIR analysis confirms intermolecular interactions between gelatin and OCMC through hydrogen bonding, successful genipin-induced crosslinking forming a stable network, and physical encapsulation of curcumin and retinyl palmitate within the hydrogels.

**Figure 2:**
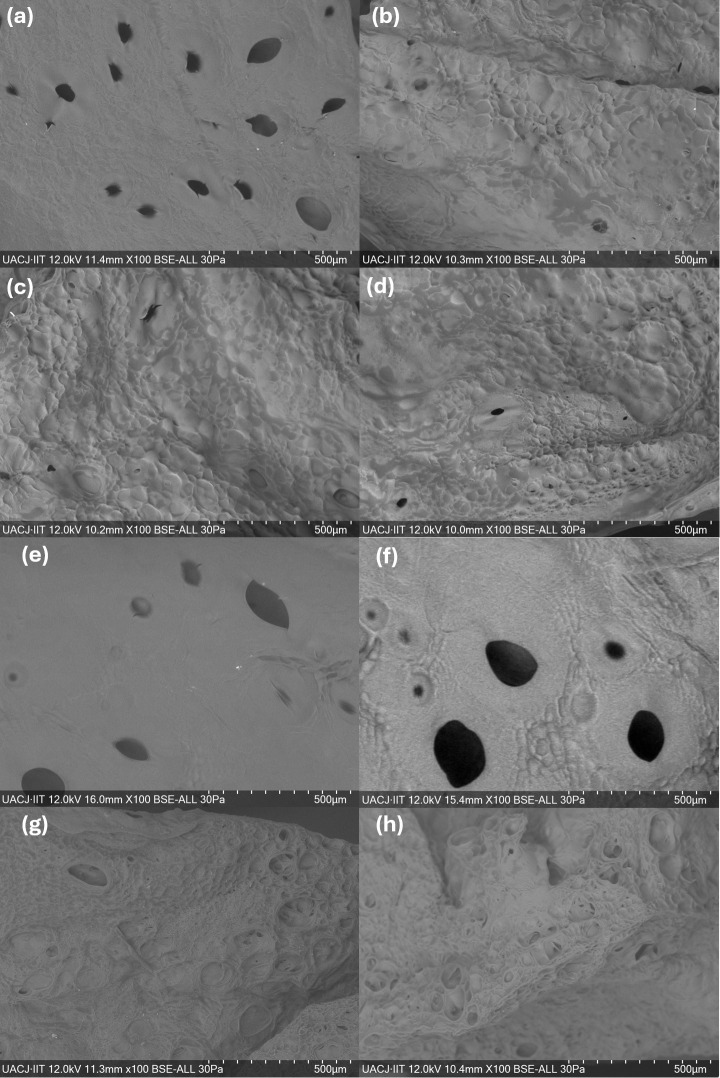
Micrographs of the hydrogels obtained at ×100 in BSE mode, (**a**) OVControl, (**b**) OV0.5R, (**c**) OV0.8R, (**d**) OV1.0R, (**e**) OV-Cur, (**f**) OV-0.5R-Cur, (**g**) OV-0.8R-Cur, and (**h**) OV1.0R-Cur.

Figure 2 shows the micrographs obtained from the SEM characterization of the hydrogels. Figure 2a shows the surface morphology of the OV-Cur hydrogel, where a closed polymer matrix with large pores of 101.45 ± 29.97 nm is visible. A similar surface morphology is observed in the OV-Cur hydrogel (Figure 2e), with large pores of 125.35 ± 42.97 nm. For the hydrogels containing retinyl palmitate, a decrease in pore size is observed, along with an increase in surface roughness and the appearance of smaller pores (Figure 2b–d). In the case of hydrogels with retinyl palmitate and curcumin, a similar trend to the previous ones is observed, showing an increase in surface roughness and the appearance of small, interconnected pores (Figure 2f–h).

**Figure 3:**
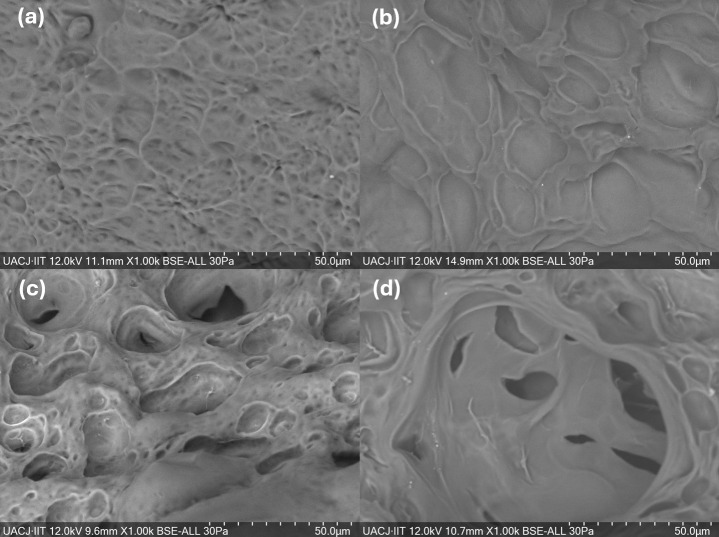
Micrographs of the hydrogels obtained at ×1000 in BSE mode, (**a**) OVControl, (**b**) OV-Cur, (**c**) OV1.0R, and (**d**) OV1.0R-Cur.

Figure 3 shows micrographs of the hydrogels at 1000× magnification. In the case of the OVControl (Figure 3a) and OV-Cur (Figure 3b) hydrogels, some surface roughness is observed, but without interconnected pores, resulting in a closed polymer matrix. However, upon adding retinyl palmitate to the polymer matrix, pores of approximately 23.12 ± 9.09 nm appear in the OV1.0R hydrogel (Figure 3c) and 17.68 ± 5.65 nm in the OV1.0-Cur hydrogel (Figure 3d), along with an increase in surface roughness. Pore size and distribution have a significant influence on the physicochemical properties of the hydrogel. For example, in the case of swelling, the polymer network requires interconnected pores for the swelling process to be rapid. Furthermore, in drug delivery systems, the porosity of a hydrogel controls the drug release profile, since the movement of these molecules is influenced by the pore size of the hydrogel [27]. Therefore, it can be seen that increasing the porosity of hydrogels containing retinyl palmitate helps to increase the porosity of interconnected pores on the surface, which can lead to an increase in the swelling properties and drug release of the system.

### 2.2. In Vitro Swelling and Degradation of Hydrogels

**Figure 4:**
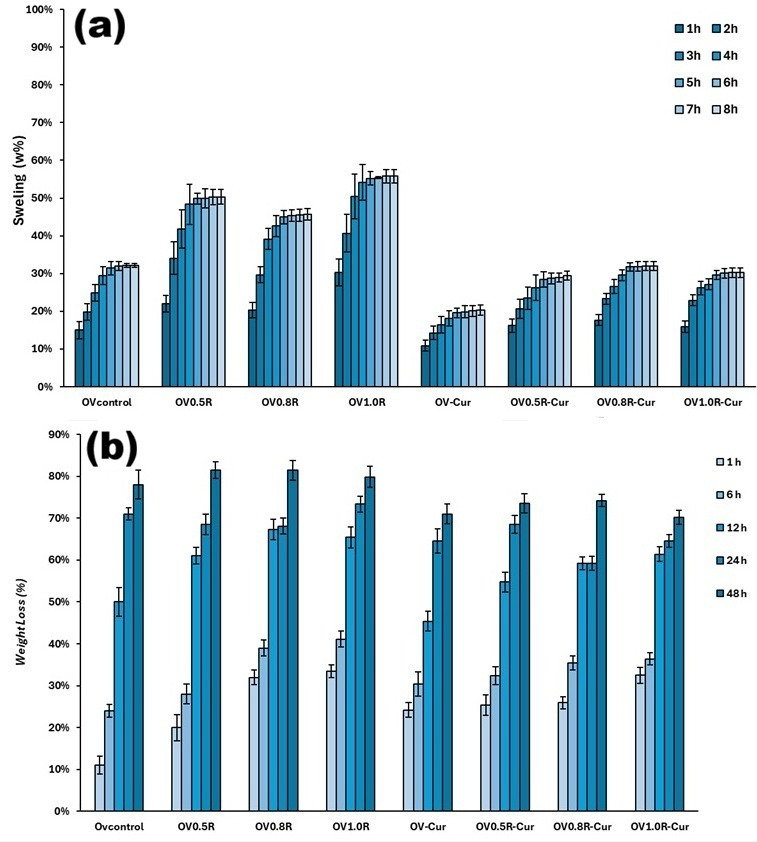
(**a**) Graph of the percentage of swelling of hydrogels at different times, (**b**) Graph of weight loss of hydrogels during the in vitro degradation test.

**Figure 5:**
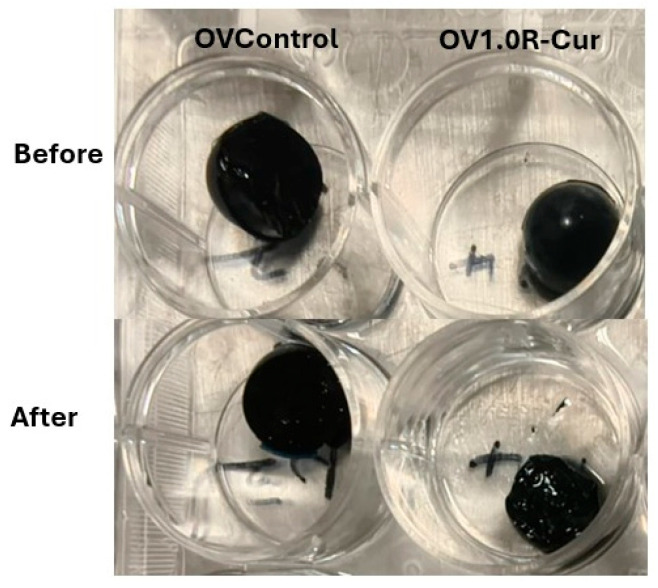
Representative images of ovules before and after swelling test, showing the macroscopic appearance and structural integrity of OVcontrol and OV1.0R-Cur formulations.

Figure 4a shows the results of the swelling test, revealing a gradual increase in the percentage of swelling up to 5 h in all samples. After this period, the swelling stabilized. The addition of PR resulted in a significant increase in swelling, reaching 32.16% ± 1.01% for the OVcontrol sample and 55.81% ± 2.05% for OV1.0R at 8 h. Conversely, a decrease in the percentage of swelling was observed upon incorporation of curcumin into the polymer matrix, yielding 20.28% ± 1.01% for the OV-Cur sample and 30.27% ± 1.08% for OV1.0R-Cur. To complement these quantitative results, Figure 5 presents representative photographs of the ovules before and after the swelling assay. The images confirm the macroscopic integrity of the systems and visually evidence the dimensional changes associated with water uptake, supporting the swelling behavior observed in Figure 4a.

In drug delivery systems, it is crucial to control drug release rates, mechanical stability, and hydration, all of which are related to swelling. A lower swelling percentage helps control drug release because it limits the movement of polymer chains and decreases pore diameters, resulting in sustained release over a longer period. It has been found that incorporating genipin as a crosslinker in OCMC and gelatin hydrogels significantly reduces the swelling percentage. For example, in carboxymethyl chitosan hydrogels with genipin, increasing the amount of genipin decreases the hydrogel swelling percentage [28]. This effect is also observed in gelatin and genipin hydrogels, where the incorporation of genipin decreases the swelling percentage [29]. A previous study found that incorporating curcumin into polymeric hydrogels reduces the swelling percentage because curcumin is a hydrophobic molecule, decreasing the hydrophilicity and water absorption of the hydrogel and acting as an additional crosslinking agent [30]. This study shows that incorporating curcumin into hydrogels significantly reduces swelling percentage. The OV-Cur sample exhibited the lowest swelling percentage at the end of the study, and the OV0.5R-Cur, OV0.8R-Cur, and OV1.0R-Cur hydrogels also showed a decrease in swelling compared to hydrogels without curcumin.

One strategy for regulating drug release in hydrogels is to control the degradation of the polymer matrix. During degradation, the mesh size increases, leading to greater diffusion of the drug into the medium. Hydrogel degradation is primarily caused by hydrolysis or enzymatic activity, through the rupture of the main polymer chain or the crosslinking agents. The most common way to analyze the degradation of a biomaterial is by measuring polymer mass loss, which can occur through volumetric or surface erosion. Mass loss can be controlled by crosslinking the hydrogel’s polymer network with crosslinking agents, as this reduces water ingress into the hydrogel, halting surface erosion and resulting in prolonged drug release [31]. Figure 4b shows the results obtained for hydrogel degradation over a period of 1 to 48 h. From the first hour, a significant increase in the degradation of the hydrogels containing retinyl palmitate and curcumin was observed. This increased degradation in the first hours of the study can be attributed to the porosity observed on the surface of the hydrogels, which increases with the incorporation of retinyl palmitate. However, it was observed at 24 h that all samples were between 65 and 73% degradation, and at the end of the test, the hydrogels with curcumin obtained a slightly lower degradation than those containing only retinyl palmitate, obtaining 78.00 ± 3.10% for the OVcontrol hydrogel, 81.50 ± 2.80%, 81.40 ± 2.40% and 79.80 ± 1.90% for the OV0.5R, OV0.8R and OV1.0R hydrogels, and 71.00 ± 2.10%, 73.50 ± 2.20%, 74.20 ± 1.40% and 70.14 ± 1.90% for the OV-Cur hydrogels. OV0.5R-Cur, OV0.8R-Cur, and OV1.0R-Cur, respectively, at the end of the study. Although degradation was observed from the first hours of the study, all samples showed stepwise degradation, maintaining their structure throughout the study, which may help maintain prolonged drug release for up to 48 h.

### 2.3. Contact Angle

**Figure 6:**
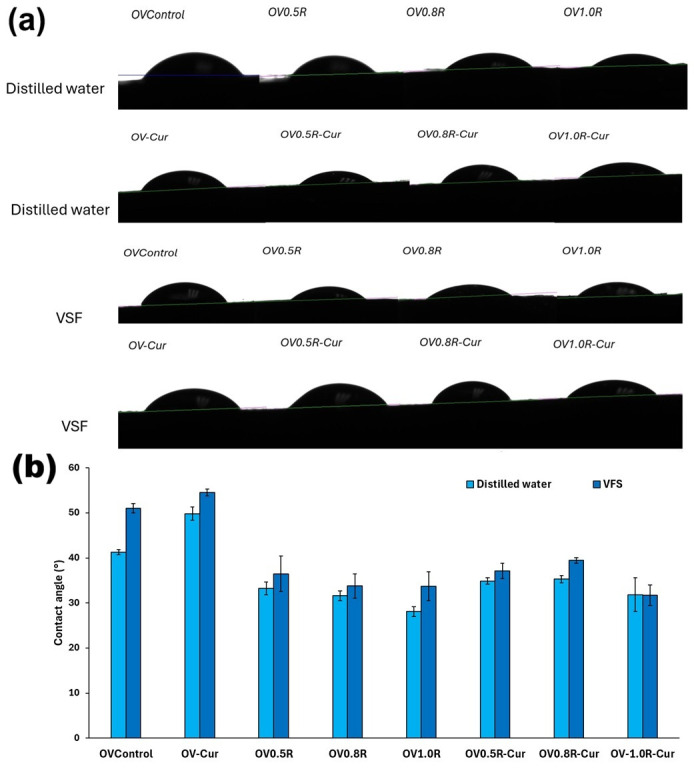
Contact angle of the hydrogels; (**a**) images of the drop on the different samples during the contact angle test, and (**b**) Graph of the contact angles obtained for the different formulations of the hydrogels using distilled water and VFS.

The contact angle test was performed to determine the hydrophilicity of the ovules in neutral solutions (distilled water) and in acidic environments such as the Vaginal Fluid Simulant (VFS). During the test, if the contact angles obtained are less than 90°, it indicates that the hydrogels have hydrophilic properties. This property can predict the wettability, adhesion, and solubility of the materials in the vaginal environment [32]. Figure 6a shows images of the droplets on the surface of the hydrogels and the contact angles of the ovule formulations. The control sample (OVcontrol) showed contact angles of 41.3 ± 0.6° and 51.1 ± 1.05° for distilled water and VFS, respectively, as shown in the graph in Figure 6b. When retinyl palmitate was added to the ovules, a significant decrease was observed, obtaining angles from 33.2 ± 1.44° to 28.1 ± 1.1° with distilled water, and from 36.5 ± 3.9° to 33.7 ± 3.2° for VFS, showing a decrease in the contact angle when increasing the amount of PR and in distilled water. When curcumin is incorporated into the ovules, the contact angle increases, as can be observed in OV-Cur, obtaining 49.8 ± 1.4° and 54.6 ± 0.7° for distilled water and VFS, respectively, and for OV0.5R-Cur, OV0.8R-Cur, and OV1.0R-Cur, results range from 34.9 ± 0.7° to 31.8 ± 3.7° for distilled water and from 37.1 ± 1.7° to 31.8 ± 2.2° for VFS. Although there is an increase in the contact angle in the formulations with curcumin, all exhibit hydrophilic properties, since the contact angles are below 90°.

The increased hydrophobicity of materials similar to curcumin has been observed in several previous studies. For example, chitosan/gelatin suppositories treated with curcumin and gemcitabine showed an increased contact angle of 20.8° to 26.9° compared to the control suppository, using distilled water during the test [19]. This is attributed to the hydrophobic properties of curcumin and its limited interaction with water. In hydroxypropyl methylcellulose (HPMC) and polyethylene glycol (PEG) 400 films, a significant decrease in the contact angle with VSF solution was observed upon the addition of retinyl palmitate to the films [33]. Previous studies have shown that the contact angle of mucoadhesive polymeric materials changes depending on the type of medium used during the contact angle test [34,35]. In this study, a slight increase in the contact angle with the VSF was observed in almost all samples; however, all were below 90°, demonstrating that the ovules are hydrophilic in acidic pH, such as in the vaginal environment.

### 2.4. Mucoadhesiveness (Glide Test)

One of the main objectives of drug delivery systems for vaginal treatments is to remain fixed at the site where localized drug release and/or absorption is required. Mucin is a glycoprotein that constitutes most of mucus, so the use of mucoadhesive polymers that bind to mucin or epithelial surfaces must be considered. In summary, mucoadhesion occurs in two stages. The first stage involves wettability, swelling, and hydration of the polymer, which ensures direct contact with the mucosal layer. In the second stage, hydration of the polymer system allows the release of mucoadhesive particles and a firmer bond with the mucin through the formation of Van der Waals interactions or hydrogen bonds [36]. Table 1 shows the results of the slippage (mucoadhesion) test on vaginal tissue of the ovules. It was observed that the OVcontrol ovule began to slide 2 mm at 60 min of the test, and this sliding increased to 6 mm at 90 min, remaining at that level until the end of the test. For the OV-cur ovule, the slide was greater and faster, reaching 4 mm at 60 min and increasing to 7 mm at 210 min. For the ovules treated with retinyl palmitate, a significant decrease in sliding was observed, with a sliding of 1 mm at 120 min for the OV0.5R sample. At 150 min, all ovules treated with retinyl palmitate showed a slide of 1 mm, which remained at that level until the end of the test. In the case of the suppositories containing retinyl palmitate and turmeric, a significant decrease in displacement was observed compared to the OV-Cur suppository. Although a displacement of 1 mm was obtained at 30 min in all samples, they maintained a displacement of 3 mm at the end of the test. Therefore, it can be concluded that the use of PR improved the mucoadhesiveness of the suppositories. Polymers such as gelatin and chitosan, and their derivatives, have proven mucoadhesive properties. This property is attributed to the presence of hydroxyl, carboxyl, and amino groups in their structure, which produce intermolecular interactions with the mucin of the vaginal mucosa. Several studies have confirmed this property, for example, in gelatin and chitosan suppositories with curcumin and gentanicin, where an accelerated mucoadhesiveness test on porcine vaginal tissue showed no slippage during the 2 h test [19]. These types of polymer systems have also demonstrated good mucoadhesive results on other types of mucosal tissues, such as chitosan and maleic acid hydrogels, where a detachment test was performed on porcine ocular mucosa [37].

### 2.5. In Vitro Drug Release

The administration of curcumin in cancer treatments has been challenging in recent years due to its poor absorption, rapid metabolism, and inadequate oral bioavailability. Furthermore, curcumin tends to degrade through hydrolysis, oxidation, and photodegradation. Therefore, the use of biomaterials such as capsules, hydrogels, films, and nanoparticles for the targeted and prolonged release of curcumin in the target tissue has been explored. Chitosan derivatives, such as OCMC, produced through carboxymethylation, have been used in the fabrication of drug delivery systems. This is because OCMC exhibits a pH-dependent release mechanism; at acidic pH, swelling and faster release occur due to the amino groups present in its chains, which become protonated in acidic environments, creating a repulsion between positive charges. Conversely, in alkaline solutions, OCMC matrices do not swell, producing a slower release, which makes them ideal polymers for drug delivery systems [38].

**Figure 7:**
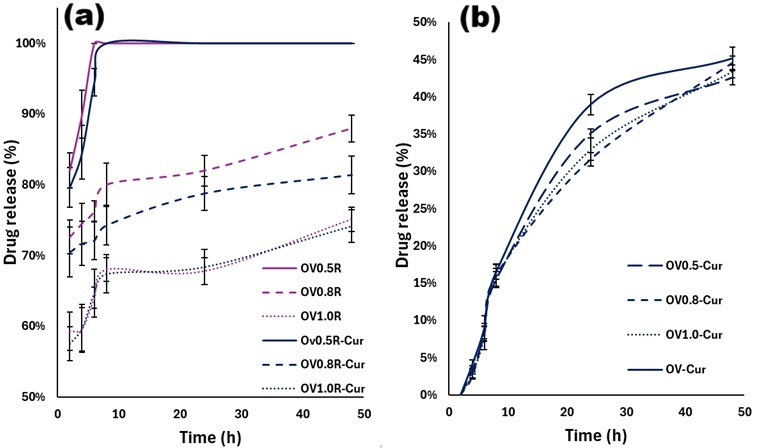
Drug release profile for the (**a**) palmitate retinyl and (**b**) curcumin from hydrogel samples.

Figure 7 shows the release test results for retinyl palmitate and curcumin in the different hydrogel formulations. In the case of retinyl palmitate (Figure 6a), the OV0.5R and OV0.5R-Cur hydrogels showed 100% release within the first few hours of the study, indicating that the addition of curcumin did not significantly affect palmitate release at low concentrations. However, increasing the concentration of retinyl palmitate in the hydrogels resulted in a lower release percentage. At 48 h, the release rates for the OV0.8R and OV0.8R-Cur samples were 87.95 ± 1.90% and 81.40 ± 2.70%, respectively, and for the OV1.0R and OV1.0R-Cur samples, 75.12 ± 1.70% and 74.20 ± 2.30%, respectively. Other studies have found rapid release of retinyl palmitate in polymer films, with complete release in less than 1 h [33]. This suggests that suppositories with higher concentrations of retinyl palmitate help achieve slower release, resulting in prolonged treatment.

In the case of curcumin release (Figure 7b), a lower and slower release was observed compared to retinyl palmitate, with release percentages at 48 h of 45.14 ± 0.98%, 42.56 ± 0.87%, 44.58 ± 1.50%, and 43.41 ± 0.89% for samples OV-Cur, OV0.5R-Cur, OV0.8R-Cur, and OV1.0R-Cur, respectively. The incorporation of retinyl palmitate into the hydrogel caused a slight decrease in curcumin release only 24 h, with no significant effect at other release times, suggesting that retinyl palmitate does not affect curcumin release. These results suggest that curcumin is released in a controlled manner.

Several studies have shown that the use of chitosan, along with its derivatives, helps delay drug release compared to other polymers. For example, in sodium alginate-chitosan hydrogels, slower release was observed in hydrogels containing a 1:4 ratio with a predominance of chitosan, compared to the 4:1 ratio with a predominance of sodium alginate. These results have been attributed to a stronger interaction between curcumin and chitosan than with sodium alginate [39]. Another study found that curcumin is released more effectively at pH 5.4 than at neutral pH, possibly promoting more effective targeting of tumor cells. This result was obtained with a chitosan-based niosomal hydrogel containing polyethylene glycol and halloysite nanotubes with curcumin [40]. Finally, a recent study found that the release of curcumin in gelatin/chitosan ovules resulted in a 76.5% release of curcumin at 8 h [19], which was higher than that found in this test. This suggests that the incorporation of genipin into the gelatin and OCMC polymer matrix, along with the reduction in swelling, helped to delay the release of curcumin, allowing for a controlled and prolonged release of the hydrogels for up to 48 h.

Kinetic analysis (Table 2) showed that the release behavior was strongly dependent on both the formulation composition and the incorporated active compound. For curcumin-loaded ovules, the Higuchi model provided the best overall fit, with high correlation coefficients (R^2^ = 0.95–0.97), indicating that diffusion through the hydrated matrix is the dominant release mechanism. This was consistent across all curcumin formulations, suggesting a relatively homogeneous dispersion of the drug and a stable diffusion pathway within the polymeric network [41,42].

In contrast, the Korsmeyer–Peppas model also showed acceptable fits for curcumin systems (R^2^ ≈ 0.85–0.90), with *n* values around or slightly above 1.0, indicating a deviation from simple Fickian diffusion and suggesting a contribution of polymer relaxation and swelling-controlled transport (anomalous or Case II transport behavior) [43].

For retinyl palmitate formulations, the kinetic behavior was more heterogeneous. While some samples showed moderate to high fits with the Higuchi model (R^2^ ≈ 0.85–0.97), other formulations exhibited very low correlation coefficients (e.g., R^2^ = 0.39 and 0.53), indicating that in these cases the release process does not follow a purely diffusion-controlled mechanism. This poor fitting can be attributed to the physicochemical characteristics of retinyl palmitate, including its high lipophilicity and stronger interactions with the semi-solid ovule matrix, which likely lead to non-uniform distribution, partitioning limitations, and possible retention within hydrophobic domains of the formulation. These effects result in a release profile that deviates significantly from the assumptions of the Higuchi model, which presumes a homogeneous matrix and constant diffusivity [41,42,44].

The Weibull model provided a more robust description of the release behavior across formulations, with R^2^ values generally between 0.87 and 0.96 for curcumin and retinyl palmitate systems. The β parameter values close to or slightly above 1 suggest a combined mechanism involving diffusion coupled with swelling and structural relaxation of the polymeric network [45].

Overall, although diffusion remains the primary mechanism in curcumin-loaded systems, the retinyl palmitate formulations exhibit a more complex release behavior that cannot be adequately described by a single classical kinetic model, reflecting the combined influence of drug lipophilicity, matrix heterogeneity, and polymer–drug interactions.

### 2.6. In Vitro Cell Viability

Several studies have demonstrated the cytotoxic effect of curcumin against different cancer cell lines. For example, a toxic effect on MCF-7 breast cancer cells, with cell viability below 70%, has been reported at concentrations of 20 µM after 24 h of exposure. This effect has been associated with a reduction in H3 glutathionylation, leading to inhibition of cell proliferation through G2/M cell cycle arrest [46]. Similarly, dose-dependent antitumor activity has been reported in MDA-MB-231 and MCF-7 breast cancer cells, where increasing curcumin concentrations significantly reduced cell viability compared to the control [47].

**Figure 8:**
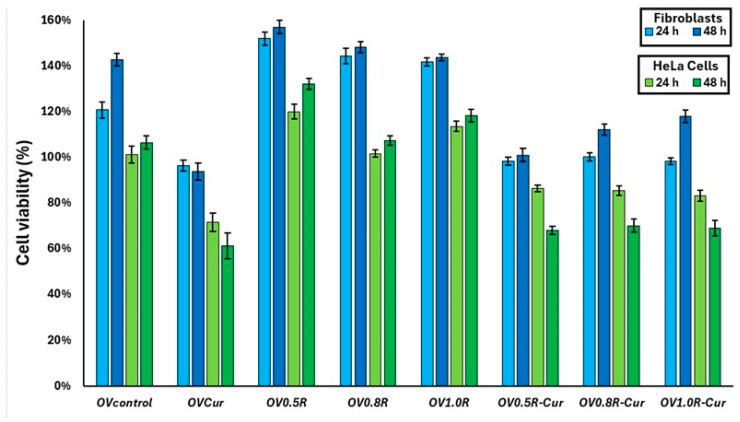
Viability assays performed on HeLa and Fibroblasts cells treated with the different hydrogel formulations.

In the present study, the OVcontrol formulation showed no cytotoxicity toward either cell line, with cell viability values of 109.40% and 106.40% after 48 h for fibroblasts and HeLa cells, respectively. In contrast, the OVCur formulation significantly reduced cell viability, reaching 71.95% in fibroblasts and 61.25% in HeLa cells (Figure 8). These results demonstrate that the curcumin released from the ovules exerted a cytotoxic effect on both cell lines, which was more pronounced in HeLa cells, suggesting preferential activity against cancer cells. Previous studies have shown that controlled-release curcumin systems can improve the selective cytotoxicity of the compound. For instance, curcumin-loaded iron oxide nanoparticles functionalized with folic acid exhibited enhanced cytotoxicity toward HeLa cells compared to L929 fibroblasts, whereas free curcumin did not show this selectivity [48].

The ovule formulations containing retinyl palmitate alone produced a considerable increase in cell viability in both cell lines. In fibroblasts, after 48 h of incubation, viability values of 157.04 ± 2.8%, 148.21 ± 2.50%, and 143.68 ± 1.40% were obtained for OV0.5R, OV0.8R, and OV1.0R, respectively. Although a slight decrease in viability was observed with increasing retinyl palmitate concentration, all formulations showed substantially higher viability than the control. This effect may be related to the known role of retinoids in promoting cell proliferation, tissue regeneration, and extracellular matrix synthesis. For the OV0.5R-Cur, OV0.8R-Cur, and OV1.0R-Cur formulations, fibroblast viability decreased compared to the formulations without curcumin; however, viability remained above 98% at both incubation times, indicating good cytocompatibility toward healthy cells. In contrast, HeLa cell viability decreased to 68.01 ± 1.70%, 70.20 ± 2.90%, and 69.02 ± 3.50%, respectively, demonstrating a selective cytotoxic effect against cancer cells.

Retinyl palmitate has been reported to regulate several biological pathways associated with tissue repair, including inflammatory signaling, collagen synthesis, apoptosis, and cell migration [49]. Previous studies have also demonstrated that incorporation of retinyl palmitate into biomaterials improves epithelial cell viability; for example, nanofibrous cellulose acetate scaffolds containing retinyl palmitate showed higher cell viability than scaffolds without the compound [11]. In addition, retinyl palmitate has been reported to reduce oxidative stress and cytogenetic damage when combined with antineoplastic drugs such as cyclophosphamide and doxorubicin, thereby decreasing the adverse effects associated with chemotherapy [50]. Antitumor effects have also been reported in vivo, where retinyl palmitate administration reduced preneoplastic skin alterations induced by 7,12-dimethylbenz[α]anthracene (DMBA) [51]. Furthermore, increased serum retinol concentrations following retinyl palmitate administration have been associated with suppression of tumor growth and adhesion in NOZC-1 xenograft mouse models [52]. Collectively, these findings support the role of retinyl palmitate as a bioactive compound capable of enhancing cytocompatibility while contributing to antitumor activity when combined with curcumin.

## 3. Conclusions

In summary, it was found that gelatin/OCMC ovules crosslinked with genipin, containing curcumin and different concentrations of retinyl palmitate, possess promising properties for use as sustained-release drug delivery systems due to their suitable swelling, degradation, and adhesive characteristics. Furthermore, release studies demonstrated that ovules containing higher concentrations of retinyl palmitate (OV0.8R-Cur and OV1.0R-Cur) exhibited prolonged release of both compounds for up to 48 h, which is desirable for sustained drug delivery applications. The kinetic analysis showed that the Weibull and Higuchi models best described the release profiles, indicating that diffusion was the predominant mechanism controlling the release of the active compounds from the ovules. Although the degradation and swelling in vitro results provided insight into how the ovules may behave under vaginal conditions, additional studies could further improve the understanding of their performance. Mechanical characterization tests could provide important information regarding their structural integrity, resistance to handling, and suitability for vaginal administration.

The in vitro cell viability study demonstrated that retinyl palmitate helped protect fibroblasts from the cytotoxic effect of curcumin while simultaneously enhancing the cytotoxic effect against HeLa cells. Additionally, increasing the concentration of retinyl palmitate in the polymeric matrix affected HeLa cell viability, with values below 70% observed for the OV1.0R-Cur sample after 48 h, without causing toxicity to fibroblasts. Overall, these results suggest that ovules containing retinyl palmitate and curcumin show promising in vitro potential as a localized and sustained delivery system. However, these findings are preliminary, and further in vivo studies are necessary to confirm their therapeutic efficacy and to fully support their potential application in cervical cancer treatment.

## 4. Materials and Methods

### 4.1. Synthesis of O-Carboxymethyl Chitosan (OCMC)

The synthesis of OCMC was carried out via a carboxymethylation reaction of chitosan in an alkaline, alcoholic medium. A total of 5 g of medium molecular weight chitosan and ≥75% deacetylated (Sigma Aldrich, St. Louis, MO, USA C3646) was weighed, moistened with 50 mL of isopropanol (99% purity, Desarrollo de Especialidades Químicas S.A. de C.VGarcia, Nuevo León,, Mexico), and stirred for 30 min at room temperature to facilitate solvent penetration into the polymeric structure. A 40% (*w*/*v*) sodium hydroxide (NaOH, Macron Fine Chemicals 7708-10, Radnor, PA, USA ) solution was prepared by dissolving 32 g of NaOH in 50 mL of alcohol. This solution was added gradually to the reaction system in four equal aliquots, with 15 min intervals between additions, while maintaining constant stirring. Subsequently, a solution of monochloroacetic acid (25.5 g of the reagent in 100 mL of isopropanol) was prepared and added in five equal portions, at 1 min intervals, while maintaining the reaction under constant stirring at room temperature. The reaction mixture was kept under constant stirring for a period of 24 h to allow for the substitution of carboxymethyl groups onto the chitosan structure and the formation of OCMC. Upon completion of the reaction time, the process was halted by the addition of 70% ethyl alcohol, and the resulting product was isolated via filtration. The obtained solid was washed repeatedly with ethyl alcohol to remove residual salts and excess reagents. The pH of the material was adjusted to 7 using a 2% acetic acid solution. Finally, the resulting OCMC was dried under vacuum overnight.

### 4.2. Fabrication of Hydrogels in the Form of Ovules

For the control ovule (Ovcontrol), an aqueous solution was prepared with 0.05 g of OCMC in 5 mL of distilled water, maintaining constant agitation until complete dissolution. Subsequently, 1 g of gelatin (Sigma Aldrich, St. Louis, MO, USA, G9382) was added. The polymeric solutions were tempered at 50 °C to facilitate the dissolution of the gelatin. Cross-linking of the system was carried out by adding 0.01 g of genipin (Challenge Bioproducts Co., Douliu City, Taiwan). The mixture was kept under agitation until homogeneous solutions were obtained. For the ovules containing retinyl palmitate, three formulations were prepared: 0.5% w/w (OvR0.5), 0.8% *w*/*w* (OvR0.8), and 1.0% *w*/*w* (OvR1.0). Finally, for the curcumin ovules, 250 µg of curcumin (Sigma Aldrich, St. Louis, MO, USA, C7727) was incorporated into each of the retinyl palmitate formulations (Gelpharma, Guadalajara, Mexico). The mixtures were carefully poured into ovule-shaped silicone molds, ensuring a uniform volume in each cavity. Finally, the molds were placed under refrigeration for 24 h to allow for solidification and the progression of the cross-linking process. After this period, the ovules were removed from the molds and stored under controlled conditions until subsequent characterization. Table 3 shows the percentage concentrations for each of the obtained ovules.

### 4.3. Physicochemical Characterization of the Ovules

For the characterization of contact angle, swelling, degradation, in vitro drug release, and ex vivo mucoadhesiveness, a vaginal fluid simulant (VFS) was used, which was prepared in accordance with Owen’s methodology [33], consisting of distilled water (900 mL), sodium chloride (3.5 g), potassium hydroxide (1.4 g), calcium hydroxide (0.22 g), albumin (0.018 g), lactic acid (2 mL), acetic acid (1 mL), glycerol (0.16 mL), urea (0.4 g), glucose (5 g), and hydrochloric acid to adjust the pH 4.5.

#### 4.3.1. FTIR and Morphological Analysis

Characterization via FTIR was performed using a Nicolet 6700 instrumentthermo fisher scientific (Waltham, MA, USA) to determine the functional groups of the various hydrogels. The spectra were obtained from 32 scans within a range of 3800 to 800 nm. Prior to SEM analysis, hydrogel samples were frozen at −80 °C for 24 h and subsequently lyophilized for 48 h to remove water while preserving the porous network structure. The characterization was performed with a SU5000 FE-SEM,(Hitachi, Tokyo, Japan), conducted in low-pressure mode and backscattered electron (BSE) mode. The average pore size was determined from the SEM micrographs using ImageJ© software (version 1.54g). A minimum of 100 pores was measured to determine the pore size for each hydrogel.

#### 4.3.2. Contact Angle Evaluation

Films were prepared from the hydrogel formulations to obtain a flat surface for testing. Measurements were performed using a goniometer (DSA 30, Kruss, GmbH, Hamburg, Germany), employing distilled water and VFS at room temperature. The left and right contact angles were measured from the captured images, and an average was calculated to obtain the result.

#### 4.3.3. In Vitro Swelling and Degradation

For the swelling test, the hydrogels were cut to obtain samples with a diameter of 1 cm. The initial dry weights (Wd) of each sample were measured. The samples were immersed in 2 mL of VFS solution for 8 h, with their weights (Ws) measured at 1 h intervals. The following Equation (1) was used to determine the swelling percentage (S%).

(1) $$S\%=\frac{\left(Ws-Wd\right)}{Wd}\times 100$$

For the in vitro degradation test, samples with a diameter of 1 cm were cut and weighed to determine their initial weight (Wi). The samples were then placed in multi-well plates containing 2 mL of VFS solution and incubated at 37 °C and 50% relative humidity. At various time intervals, samples were removed and allowed to dry to eliminate moisture. Finally, the samples were weighed again to determine their final weight (Wf), and the percentage of weight loss (Wl%) was calculated using the following Equation (2):

(2) $$Wl\%=\frac{\left(Wi-Wf\right)}{Wi}\times 100$$

### 4.4. Drug Release Test

Hydrogel samples with a diameter of 1 cm were placed in well plates, and 3 mL of VFS was added. The drug loading percentage of curcumin in the hydrogels (OV-Cur, OV0.5R-Cur, OV0.8R-Cur and OV1.0R-Cur) was 0.024%, whereas for retinyl palmitate in the ovules, the drug loading values were 0.50% for OV0.5R and OV0.5R-Cur, 0.81% for OV0.8R and OV0.8R-Cur, and 0.98% for OV1.0R and OV1.0R-Cur. Subsequently, the samples were incubated at 37 °C and 50% relative humidity. At predetermined time intervals (0, 2, 4, 6, 8, 24, and 48 h), 0.1 mL aliquots of the release medium were withdrawn and immediately replaced with an equal volume of fresh pre-warmed medium to maintain constant volume and sink conditions, ensuring a constant release volume and maintaining the drug concentration below its saturation solubility, as previously reported for vaginal curcumin-loaded delivery systems [19,53]. For measurement purposes, ethanol was added to the resulting solutions in a 1:1 ratio to precipitate the gelatin. The solutions were analyzed at 422 nm to quantify curcumin and at 325 nm to quantify PR using a NanoDrop 2000 spectrophotometer (Thermo Scientific, Waltham, MA, USA). Calibration curves were generated to determine the concentration of both substances. Table 4 shows the linearity equation, correlation coefficient, limit of detection (LOD), and limit of quantitation (LOQ) obtained during both release tests for both drugs. The accuracy of the analytical method was determined by calculating the percentage recovery at three different concentration levels. Precision was evaluated through repeatability studies and expressed as the relative standard deviation (RSD %). Both accuracy and precision were assessed using freshly prepared samples under intraday (same day) and inter-day (three consecutive days) conditions. All measurements were performed in triplicate at each concentration level for each drug (curcumin and retinyl palmitate). According to the recovery and RSD values recorded in Table 5, all values are within the accepted range (90–107% recovery and <5.3% of RSD), guaranteeing a high level of accuracy and precision for the developed drug release method [54].

The percentages of curcumin and retinyl palmitate released were determined from the following Equation (3) [55]:

(3) $$Drug\;Release\left(\%\right)=\frac{Release\;drug\;from\;hydrogel}{Total\;amount\;of\;drug\;in\;hydrogel}\times 100$$

The experimental release data were fitted to different mathematical models to describe the release mechanism of curcumin and retinyl palmitate from the polymer matrix. The models applied were those of Higuchi, Korsmeyer-Peppas, and Weibull. The Higuchi model applies to matrix systems where release is primarily controlled by diffusion of the active compound through the polymer matrix. Equation (4) is used:

(4) $$\frac{M_{t}}{M_{\infty }}=k_{H}\surd t$$

where Mt is the amount released over time, M∞ is the total amount that can be released, and k_H_ is the Higuchi constant.

The Korsmeyer-Peppas model is used to describe anomalous or combined release mechanisms (diffusion and matrix relaxation), using the following Equation (5):

(5) $$\frac{M_{t}}{M_{\infty }}=kt^{n}$$

where k is the kinetic constant of the system, and *n* is the diffusion exponent.

Finally, the Weibull model is characterized by its ability to adjust the complete drug release profile, from 0% to 100% of the dose; Equation (6) is as follows:

(6) $$\frac{M_{t}}{M_{\infty }}=1-{\mathrm{e}}^{-{\left(\frac{t}{\tau }\right)}^{\beta }}$$

where τ is the scale parameter (release time constant), and β is the shape parameter (dispersion exponent).

### 4.5. Ex Vivo Mucoadhesion Assay

For this test, porcine vaginal tissue, obtained from the Municipal Slaughterhouse in Ciudad Juárez, Chihuahua, Mexico, was used within six hours of slaughter. The tissue was washed with a PBS solution and dissected to expose the vaginal tissue surface. Hydrogel samples were cut into circles approximately 2 cm in diameter and placed on the tissue sample at a 60° angle. Throughout the duration of the test, SVF was sprayed onto the tissue every 5 min to maintain its moisture. The test was conducted over a period of 6 h, and the slippage of the samples was measured every 30 min [33,56].

### 4.6. Analysis of the In Vitro Cytotoxic Effect

Cervical tumor cells (HeLa) and primary fibroblasts derived from Balb/c mice, obtained via enzymatic digestion with collagenase, were utilized (Research Ethics Committee Approval No.: CEI-2025-1-31). Both cell types were cultured separately in 24-well plates using Dulbecco’s Modified Eagle Medium (Sigma-Aldrich^®^, D5523, Sigma-Aldrich, St. Louis, MO, USA), supplemented with 10% fetal bovine serum (Gibco™, F2442-100, Waltham, MA, USA) and a 2% penicillin-streptomycin-amphotericin B solution (Sigma-Aldrich^®^, A5955), for 24 h prior to the study at 37 °C and 5% CO_2_. Subsequently, the various hydrogel formulations were added in triplicate, and the cells were incubated for 24 and 48 h. Untreated cells of both types served as the control group. Following the incubation periods, the supplemented culture medium was removed, and fresh culture medium containing the MTT reagent at a concentration of 5 mg/mL was added to each well; the cells were then incubated for one hour. Finally, the formazan crystals generated during the incubation period were solubilized with DMSO, and absorbance was measured at 570 nm using a microplate spectrophotometer(Benchmark Plus, Hercules, CA, USA). Cell viability % was calculated using the following Equation (7):

(7) $$\%\;cell\;viability=\left(\frac{Absorbance\;of\;treated\;cells}{Absorbance\;of\;control\;cells}\right)\times 100$$

### 4.7. Statistical Analysis

All quantitative experiments were performed using a minimum of three independent samples per hydrogel formulation, and the results are presented as mean ± standard deviation. In vitro Swelling, degradation, cytotoxicity, and contact angle data were analyzed using one-way ANOVA, followed by Tukey’s multiple comparison test. Drug release profiles were analyzed using two-way ANOVA, considering formulation and time as independent factors, followed by Tukey’s multiple comparison test. Differences were considered statistically significant at *p*-value < 0.05.

## Figures and Tables

**Table 1:** Result of the glide test of the ovules in porcine vaginal tissue by measuring the sliding.

Glide (mm)												
Sample /Time (min)	30	60	90	120	150	180	210	240	270	300	330	360
OVControl	0	2	6	6	6	6	6	6	6	6	6	6
OV0.5R	0	0	0	1	1	1	1	1	1	1	1	1
OV0.8R	0	0	0	0	1	1	1	1	1	1	1	1
OV1.0R	0	0	0	0	1	1	1	1	1	1	1	1
OV-Cur	2	4	6	6	6	6	7	7	7	7	7	7
OV0.5R-Cur	1	2	3	3	3	3	3	3	3	3	3	3
OV0.8R-Cur	1	2	3	3	3	3	3	3	3	3	3	3
OV1.0R-Cur	1	1	3	3	3	3	3	3	3	3	3	3

**Table 2:** Kinetic models fitted for each ovule formulation, including R^2^ and kinetic parameters.

Sample	Korsmeyer-Peppas				Higuchi		Weibull			
	Curcumin		Retinyl Palmitate		Curcumin	Retinyl Palmitate	Curcumin		Retinyl Palmitate	
	R^2^	*n*	R^2^	*n*	R^2^	R^2^	R^2^	β	R^2^	β
OVCur	0.9017	0.9233	--	--	0.9506	--	0.9193	1.0372	--	--
OV0.5R-Cur	0.8887	0.9605	N/A	N/A	0.9604	0.5324	0.9075	1.0649	0.842	0.5137
OV0.8R-Cur	0.852	1.0163	N/A	N/A	0.9724	0.9758	0.8769	1.1224	0.9644	0.1073
OV1.0R-Cur	0.8741	1.0069	N/A	N/A	0.9728	0.851	0.8953	1.1119	0.9195	0.1374
OV0.5R	--	--	N/A	N/A	--	0.3952	--	--	1	0.4252
OV0.8R	--	--	N/A	N/A	--	0.9419	--	--	0.9407	0.149
OV1.0R	--	--	N/A	N/A	--	0.8567	--	--	0.8738	0.1318

**Table 3:** Concentrations of the hydrogel’s components.

Sample	Gelatin	OCMC	Genipin	Retinyl Palmitate	Curcumin
Ovcontrol	94.34%	4.72%	0.94%	0%	0%
OV0.5R	93.87%	4.69%	0.94%	0.50%	0%
OV0.8R	93.58%	4.68%	0.94%	0.80%	0%
OV1.0R	93.39%	4.68%	0.93%	1.00%	0%
OV0.5R-Cur	93.84%	4.70%	0.94%	0.50%	0.02%
OV0.8R-Cur	93.56%	4.68%	0.94%	0.80%	0.02%
OV1.0R-Cur	93.37%	4.68%	0.93%	1.00%	0.02%
OV-Cur	94.32%	4.72%	0.94%	0%	0.02%

**Table 4:** Linearity equation, correlation coefficient, LOD, and LOQ of release test for curcumin and retinyl palmitate.

Drug	Correlation Coefficient	Linearity Equation	LOD (µg/mL)	LOQ (µg/mL)
Curcumin	0.9852	y = 0.0041x − 0.0906	37.61	113.99
Retinyl palmitate	0.9864	y = 0.4909x − 0.2172	141.9	430.91

**Table 5:** Results of intra-day and inter-day accuracy and precision (*n* = 3) for curcumin and retinyl palmitate.

Drug	Concentration (μg/mL)	Intra-Day			Inter-Day		
		Recovered Quantity (μg/mL)	Recovery (%)	RSD (%)	Recovered Quantity (μg/mL)	Recovery (%)	RSD (%)
Curcumin	50	50.41 ± 0.51	100.82	1.01	51.25 ± 0.85	102.50	1.65
	150	150.85 ± 0.64	100.56	0.42	152.04 ± 0.74	101.36	0.48
	250	249.26 ± 0.34	99.70	0.13	251.84 ± 1.02	100.73	0.40
Retinyl palmitate	500	501.24 ± 0.54	100.24	0.10	503.47 ± 1.54	100.69	0.30
	800	802.55 ± 0.41	100.31	0.05	803.65 ± 2.14	100.45	0.26
	1200	1204.85 ± 2.10	100.40	0.17	1206.82 ± 2.41	100.31	0.19

## Data Availability

The original contributions presented in this study are included in the article. Further inquiries can be directed to the corresponding author.

## Abbreviations

The following abbreviations are used in this manuscript:

OCMC	O-carboxymethyl chitosan
FTIR	Fourier Transform Infrared
HPV	Human papillomavirus
ROS	Reactive oxygen species
VFS	Vaginal Fluid Simulant
HPMC	Hydroxypropyl methylcellulose
PEG	Polyethylene glycol
